# Exploring the Clinicopathological Diversity in Sarcomatous Transformations in the Uterus

**DOI:** 10.7759/cureus.62671

**Published:** 2024-06-19

**Authors:** Neelayadakshi B, Sudha V

**Affiliations:** 1 Department of Pathology, Saveetha Medical College and Hospitals, Saveetha Institute of Medical and Technical Sciences, Saveetha University, Chennai, IND

**Keywords:** low-grade ess, high-grade ess, leiomyosarcoma, carcinosarcoma, spectrum of malignant uterine sarcomatous transformations

## Abstract

Introduction

Uterine sarcomas are a rare group of mesenchymal tumors. They originate either from the uterine smooth muscle or from the endometrial stroma. The spectrum of malignant uterine sarcomatous transformations includes leiomyosarcoma, low-grade endometrial stromal sarcoma (LG-ESS), high-grade endometrial stromal sarcoma (HG-ESS), undifferentiated uterine sarcoma, and carcinosarcoma. The purpose of this study is to determine the distribution of malignant uterine sarcomatous transformations, in relation to the patient’s age, epidemiological aspects, and clinical features, and to analyze the different histological types of uterine sarcomas diagnosed at our institution and thereby to quantify their frequency and determine the most common histopathological subtype occurring in the population.

Materials and methods

This was a retrospective descriptive study of 21 cases of malignant uterine sarcomatous transformations, diagnosed in Saveetha Medical College, Chennai, between January 2019 and December 2022. Data was collected from the archives of the department of pathology and from the department of medical records in the hospital. The spectrum of uterine sarcomas was analyzed in relation to the patient’s age, menopausal status, presenting symptoms, preoperative diagnosis, biopsy and frozen section reports wherever available, and histopathological reports with pathological staging.

Results

The most common age group in which uterine sarcoma was diagnosed was found to be 61-70 years. All 10 patients who were diagnosed with carcinosarcoma were aged 60 years, and all seven patients diagnosed with low-grade endometrial stromal sarcoma were less than 50 years of age. Most of the patients were postmenopausal females, except for one patient who was premenopausal. The most common histological variety found was carcinosarcoma, malignant mixed mullerian tumor (47.6%), followed by LG-ESS (33.3%), leiomyosarcoma (14.28%), and HG-ESS (4.7%).

Conclusion

Uterine sarcomas are an aggressive group of tumors having a very poor prognosis. Due to its rarity and histopathological diversity, there is a lack of consensus on the risk factors.

## Introduction

Sarcomas arise from the mesenchymal elements and are different from carcinomas, which arise from the epithelial elements in the tissue. They primarily arise from two tissues in the uterus, namely, the uterine smooth muscle and the endometrial stroma. The tumors arising due to the malignant transformation of the uterine smooth muscles are called leiomyosarcoma of the uterus. If the malignant transformation of the mesenchymal elements is associated with a benign epithelial component, it is known as adenosarcoma. However, if it is associated with a malignant epithelial component, it is known as carcinosarcoma. If there is no recognizable epithelial component associated with the malignant transformation of the mesenchymal elements, then it is known as endometrial stromal sarcoma. Exposure to radiation may increase the risk of carcinosarcoma. The time interval between radiation exposure and the development of sarcoma in the uterus can range from one to 37 years, and the type of sarcomatous transformation that predominantly occurs in the uterus postradiation exposure is carcinosarcoma [1]. Sarcomatous transformations of the uterus are rare and account for approximately 1% of all the malignancies of the female genital tract and around 5% of all uterine cancers [2,3].

Uterine sarcomas are aggressive tumors with a diverse histopathological presentation. Females with uterine sarcoma have a nonspecific clinical presentation. Typically, they present with clinical features such as abdomen pain, vaginal bleeding, and a rapidly growing mass in the pelvis. Uterine sarcomas were first classified as leiomyosarcoma, endometrial stromal sarcoma, undifferentiated sarcomas, and carcinosarcomas. However, recently, carcinosarcomas have been considered a dedifferentiated form of endometrial carcinoma. In spite of this, due to its aggressive behavior, when compared to ordinary endometrial carcinomas, carcinosarcoma is still included in many retrospective studies of uterine sarcomas, as in the 2003 WHO classification [2]. The pathogenesis of uterine sarcoma is still unclear. Mutations, amplifications, and overexpressions have been identified by genetic profiling. However, the molecular mechanisms of tumorigenesis caused by these genetic aberrations are still not fully understood. Complex chromosomal rearrangements have been identified recently by genome-wide studies as the mechanism for oncogenesis. p16 and p53, which are cell cycle regulators, are commonly overexpressed and are believed to be involved in the pathogenesis of sarcomagenesis, and hence, clinical trials are now evaluating molecular targeted therapy for the treatment of certain subtypes [3]. Due to the rarity and histopathological diversity among uterine sarcomas, there is a lack of consensus on the risk factors, resulting in the lack of optimal treatment options and poor prognosis. The aim of the present study is to determine the distribution of malignant uterine sarcomatous transformations, in relation to the patient’s age, epidemiological aspects, and clinical features, and to analyze the different histological types of uterine sarcomas diagnosed at our institution and thereby to quantify their frequency and determine the most common histopathological subtype occurring in the population.

## Materials and methods

This study was done in the department of pathology at Saveetha Medical College, Thandalam, Chennai. It is a retrospective descriptive study done on 21 patients, who were diagnosed to have sarcomatous differentiation in the uterus over a period of four years, between January 2019 and December 2022. The required data regarding uterine sarcomas was collected from the hospital’s tumor registry between January 2019 and December 2022. A complete enumeration sampling method was used, and all the histo-morphologically confirmed uterine sarcoma cases received during the period of study were included. The specimen was received in the department of histopathology, in a container filled with an adequate amount of fixative, from the operation theater. The fixative used was 10% neutral buffered formalin. The request form containing the patient’s name, age, presenting complaints with the duration of the same, clinical diagnosis, imaging findings, and other relevant clinical data; the surgical procedure done; and any significant intraoperative findings was sent along with the specimen.

The presence of the abovementioned details for each specimen was checked in the request form, before receiving the specimen in the histopathology laboratory. Each specimen was then given a unique histopathological laboratory number for identification. The specimen was then oriented and adequately fixed in 10% neutral buffered formalin. Following this, a gross examination of the specimen was done, and the representative areas were identified. Sections were then taken from the representative areas using standard guidelines. The representative sections were taken from the tumor: areas with the deepest myometrial invasion, areas of necrosis, any suspicious-looking areas, and the margins for each case. Then, these were reviewed microscopically, and the histopathological diagnosis was given with the stage of the tumor. An automated tissue processor was used to process the tissue sections. Sections were cut at a thickness of 4-5 μm, and the slides were prepared. Routine hematoxylin and eosin staining was done, and the slides were submitted for review to the pathologist. The slides were thoroughly reviewed, and the histopathological diagnosis was given for each case. Variables studied include the patient’s age, menopausal status, presenting symptoms, imaging findings, preoperative diagnosis, and histopathological diagnosis.

## Results

Age distribution

The most common age group in which uterine sarcoma was diagnosed was found to be 61-70 years (Table 1).

**Table 1:** Age distribution of females with uterine sarcoma

Age group	Percentage of females with uterine sarcoma
31-40 years	10%
41-50 years	30%
51-60 years	10%
61-70 years	50%

All 10 patients who were diagnosed with carcinosarcoma were aged 60 years and above. On the contrary, all seven patients diagnosed with low-grade endometrial stromal sarcoma (LG-ESS) were less than 50 years of age. Most of the patients were postmenopausal females, except for one patient who was premenopausal.

Histological distribution

**Figure 1:**
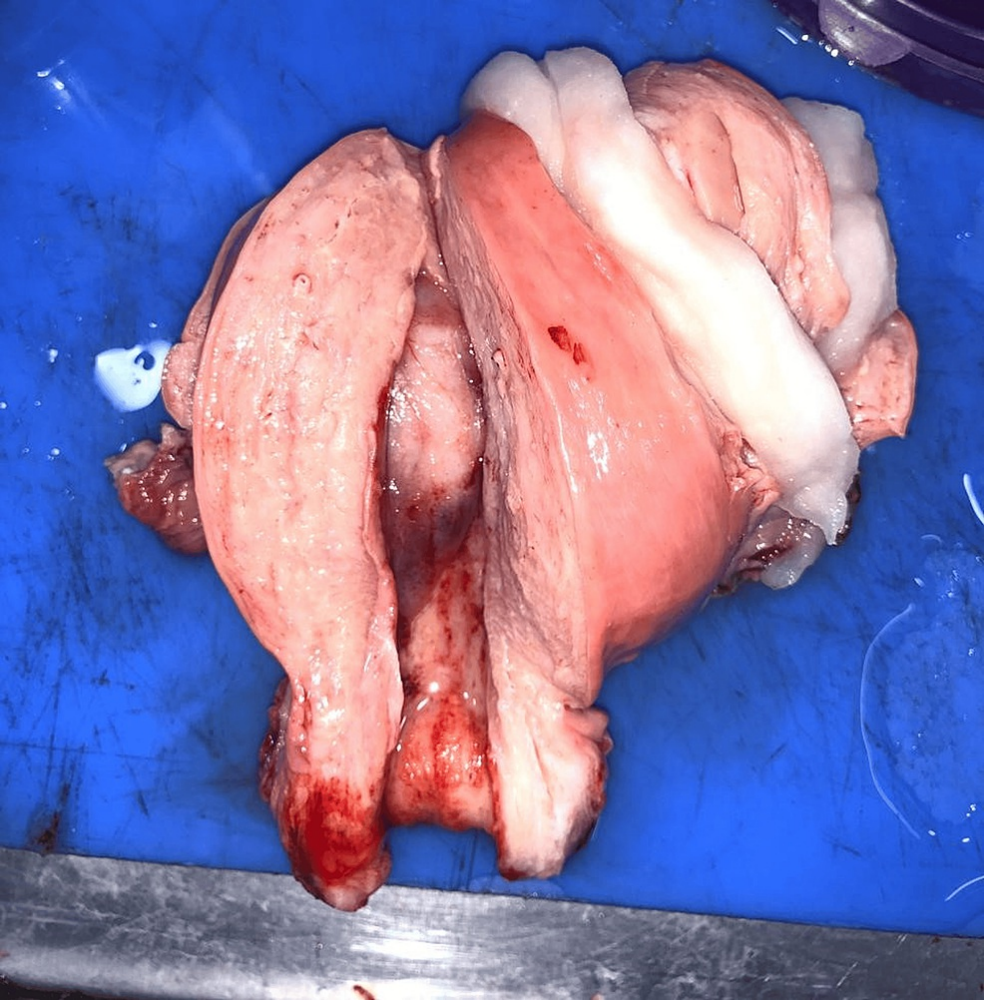
Gross specimen of carcinosarcoma The bivalving section of the uterus showed a fleshy, friable, polypoid lesion arising from the uterine fundus and was seen protruding into the endometrial cavity

**Figure 2:**
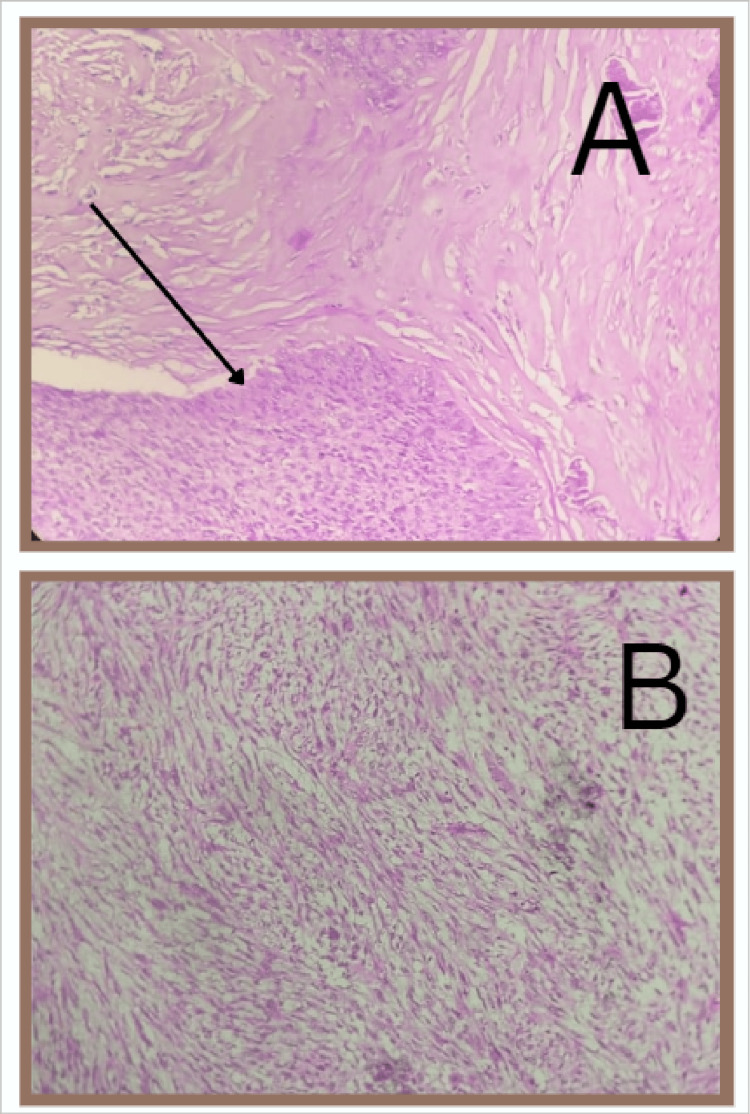
Microscopy of carcinosarcoma (20× magnification) A high-grade biphasic tumor having both carcinomatous and sarcomatous elements is seen. (A) The malignant epithelial component of carcinosarcoma (indicated by the arrow). (B) The mesenchymal component of carcinosarcoma (sarcomatous component)

**Figure 3:**
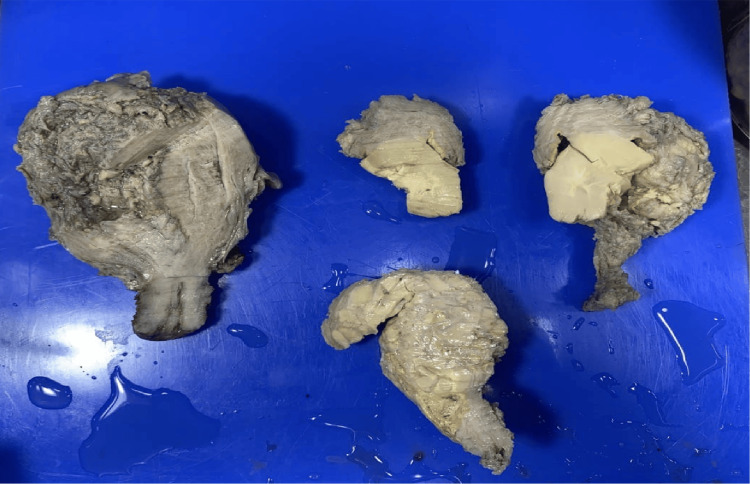
Gross specimen of endometrial stromal sarcoma The cut section of the uterus showed soft tan-yellow to tan-white nodular lesions, which were seen extending from the endometrium and invading into the myometrium (the depth of myometrial invasion was less than 50% of the myometrial thickness)

**Figure 4:**
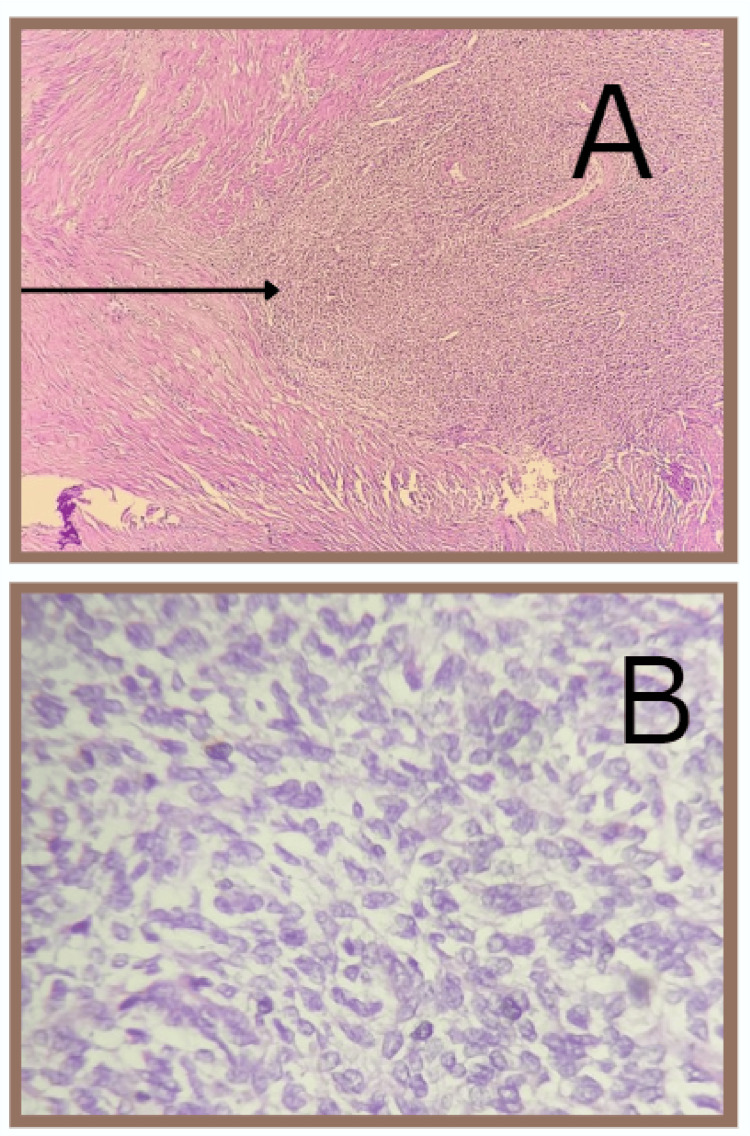
Microscopy of low-grade endometrial stromal sarcoma (A) This section shows tumor cells invading into the myometrium as tonguelike projections as indicated by the arrow (scanner view). (B) The tumor cells are monotonous oval to spindle cells having scant cytoplasm and vesicular nucleus with minimal nuclear atypia (20× magnification). Mitotic activity was less than 10 mitoses per 10 high-power fields. No necrosis was seen

The most common histological subtype seen was carcinosarcoma of the endometrium (malignant mixed mullerian tumor: 47.6%). Grossly, a fleshy, friable, polypoid lesion was seen filling the uterine cavity as seen in Figure 1, and microscopically, a high-grade biphasic tumor having both carcinomatous and sarcomatous elements was seen (Figure 2), followed by low-grade endometrial stromal sarcoma (33.3%), in which grossly, the bulky uterus had soft yellow-tan to tan-white nodular lesions, which were seen extending from the endometrium and invading into the myometrium as seen in Figure 3, where less than 50% of the myometrial thickness was involved. Microscopically, low-grade endometrial stromal tumor was seen as irregular islands of monotonous oval to spindle cells having scant cytoplasm and vesicular nucleus with minimal nuclear atypia. These tumor cells were seen invading into the myometrium as tonguelike projections, as seen in Figure 4.

**Figure 5:**
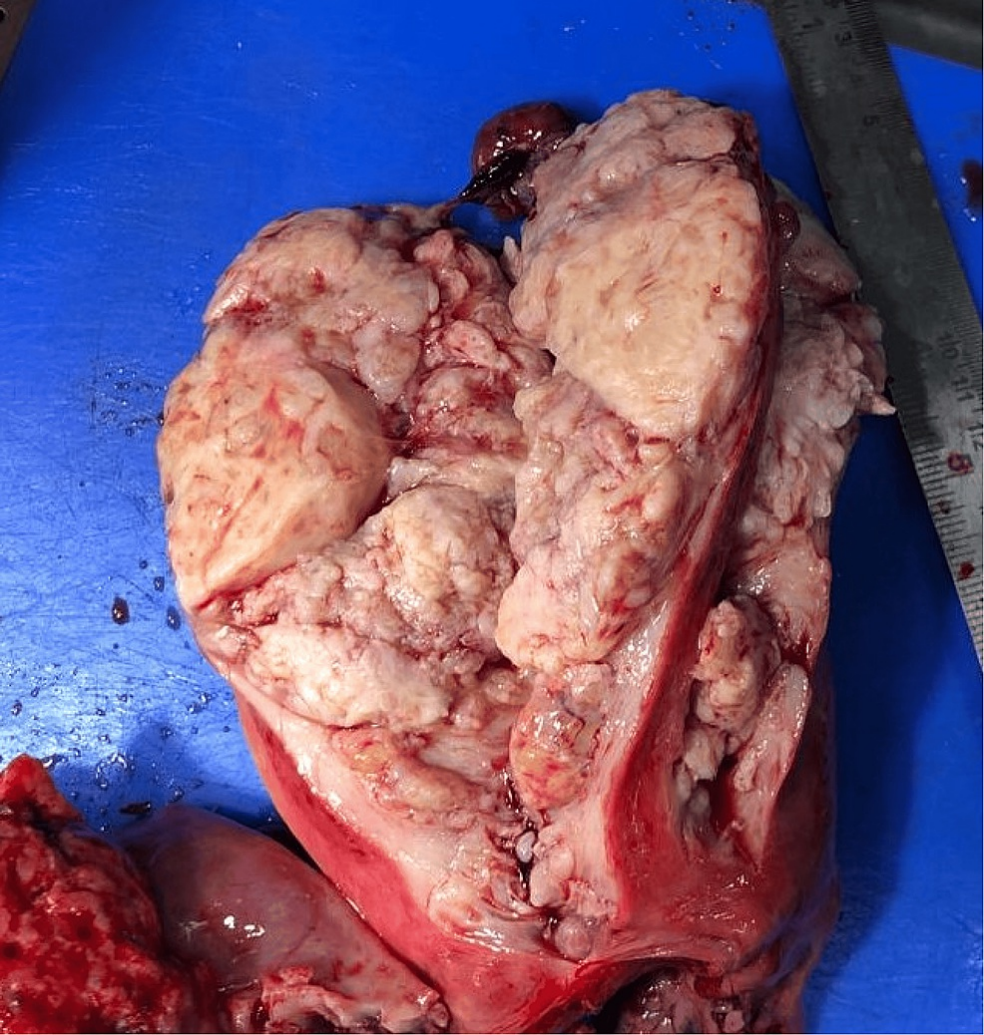
Gross specimen of leiomyosarcoma The picture shows a large, bulky, poorly defined fleshy mass involving the entire fundus and the body of the uterus. The tumor showed focal areas of necrosis and cystic changes

**Figure 6:**
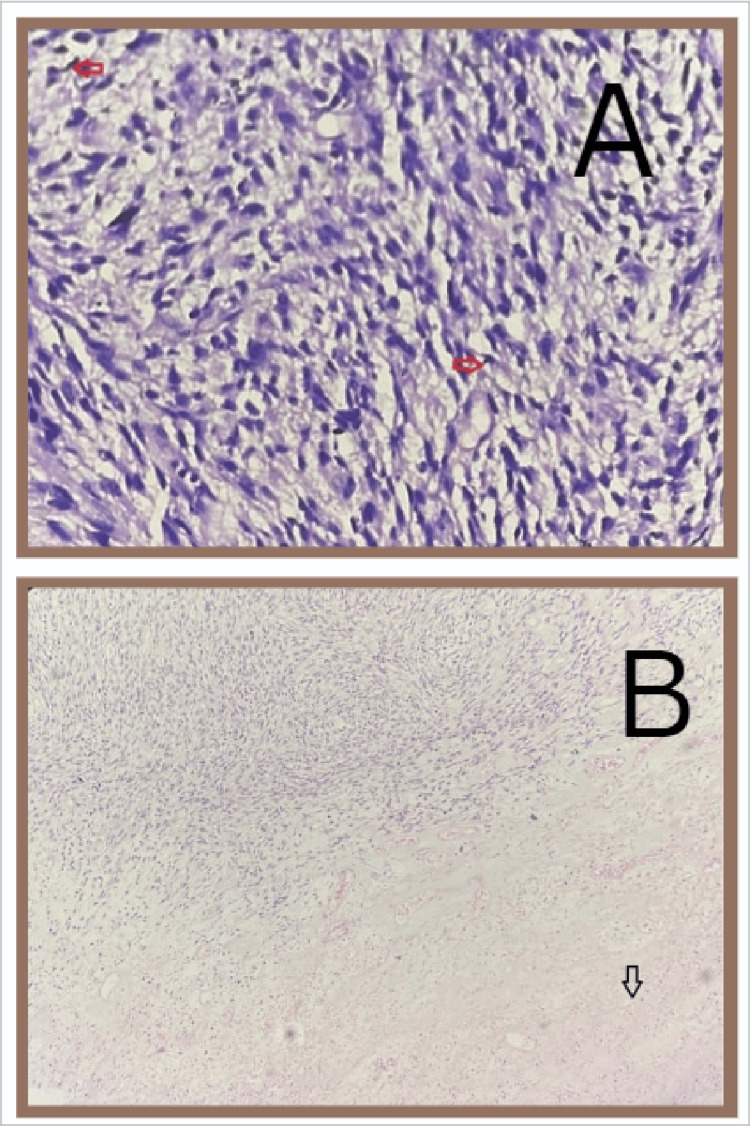
Microscopy of leiomyosarcoma (20× magnification) (A) The section shows malignant tumor cells arranged in fascicles. The tumor cells are spindle-shaped, with moderate to scant cytoplasm, showing severe nuclear atypia and a highly pleomorphic, hyperchromatic nucleus. Brisk mitosis with more than 10 mitosis per 10 HPFs was seen. The arrows indicate mitotic figures. (B) Areas of necrosis were seen, as indicated by the arrow HPFs: high-power fields

This was followed by leiomyosarcoma (14.2%), in which grossly, a large, bulky, poorly defined fleshy mass was seen in the myometrium, having necrotic areas as seen in Figure 5; microscopically, it showed a cellular neoplasm composed of malignant tumor cells arranged in fascicles; the tumor cells were spindle-shaped, with highly pleomorphic, hyperchromatic nucleus; marked cytologic atypia was seen along with areas of necrosis and having more than 10 mitosis per 10 high-power field (Figure 6) and high-grade endometrial stromal sarcoma (HG-ESS) (4.7%) (Table 2). The microscopy of the sections from the high-grade endometrial stromal sarcoma showed tumor cells arranged as nests and islands. The tumor cells had scant cytoplasm, with a round to oval nucleus, showing uniform nuclear atypia. Brisk mitosis (more than 10 mitosis per 10 high-power fields) was seen with areas of necrosis.

**Table 2:** Number of cases seen in each subtype of uterine sarcoma

Uterine sarcoma subtype	Number of cases	Percentage
Carcinosarcoma	10	47.6%
Leiomyosarcoma	3	14.28%
High-grade endometrial stromal sarcoma	1	4.7%
Low-grade endometrial stromal sarcoma	7	33.3%

All 21 patients presented with increased bleeding per vaginum and nonspecific symptoms such as abdomen pain. One patient alone had a history of passing out a mass per vaginum, which was subjected to histopathological examination (HPE) and was diagnosed as carcinosarcoma. One other patient presented with complaints of difficulty in passing urine, due to the mass effect of the tumor on the bladder, and hence was catheterized.

During the preoperative period, of the 21 cases included in the study, four cases were diagnosed as fibroid uterus, and one case was diagnosed as carcinosarcoma of the uterus preoperatively, due to the histopathological examination done for the mass expelled per vaginum. The other remaining 16 cases were given a preoperative diagnosis of endometrial carcinoma, with the help of imaging findings done preoperatively.

In the present study, 18 patients diagnosed with uterine sarcomas presented at stage I of the disease, two carcinosarcoma patients presented at stage III of the disease, and one high-grade endometrial stromal sarcoma patient presented at stage II of the disease.

## Discussion

**Figure 7:**
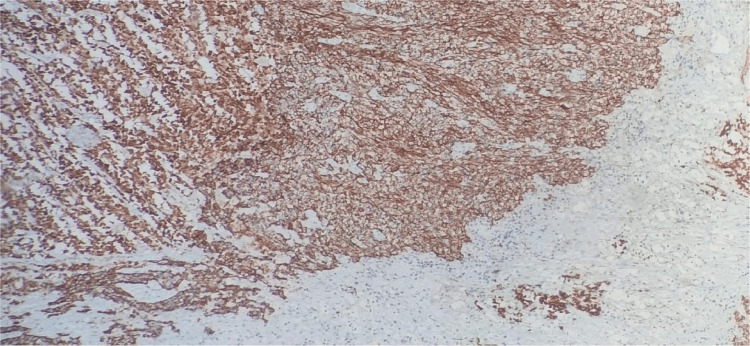
Carcinosarcoma: IHC-pan-cytokeratin positive in the epithelial component IHC-pan-cytokeratin: 80%-90% strong cytoplasmic positivity seen in the epithelial component of carcinosarcoma IHC: immunohistochemistry

**Figure 8:**
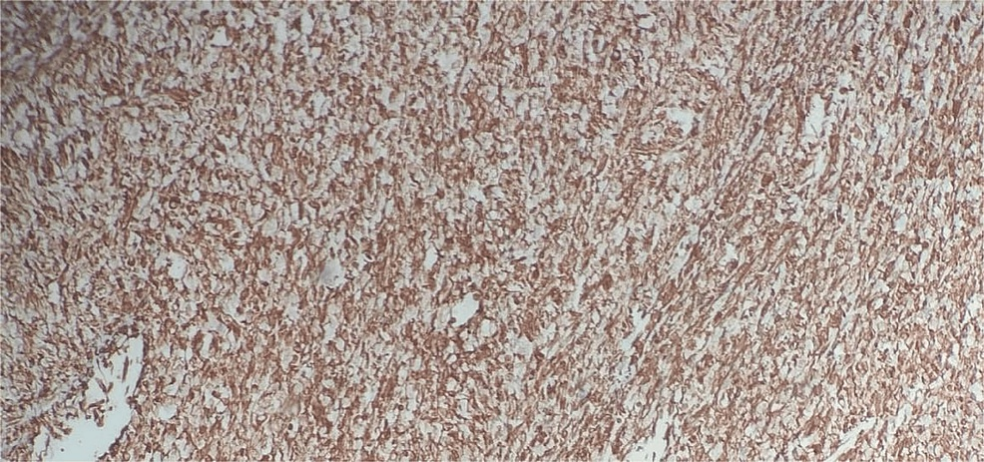
Carcinosarcoma: IHC-vimentin positive in the mesenchymal component IHC-vimentin: 90%-95% strong cytoplasmic positivity is seen in the stromal cells IHC: immunohistochemistry

Uterine sarcomas are a group of uncommon tumors that constitute less than 3% of all female genital tract malignancies and around 3%-7% of all cancers arising from the uterus [2,4,5]. They are aggressive tumors with a poor prognosis [2]. Previously, uterine mesenchymal tumors were classified either as smooth muscle neoplasms or as endometrial stromal neoplasms. The application of molecular techniques in recent times has identified many uterine lesions with unique genetic abnormalities and clinicopathological features [6]. Sarcomas of the uterus were classified into leiomyosarcomas, carcinosarcomas, high-grade and low-grade endometrial stromal sarcomas, and undifferentiated sarcomas. However, recently, carcinosarcomas have been reclassified as a metaplastic variant or a dedifferentiated form of endometrial carcinoma [2]. Carcinosarcomas are still included in most of the retrospective studies done on uterine sarcomas and also in the 2003 WHO classification, due to its very aggressive behavior when compared to routine endometrial carcinoma [2], having an overall five-year survival rate of less than 35% [7], and although some consider carcinosarcoma as a high-grade variant of carcinoma, both the National Comprehensive Cancer Network clinical practice guidelines and the new International Federation of Gynecology and Obstetrics (FIGO) staging consider carcinosarcoma under uterine sarcoma [8]; hence, we include carcinosarcoma in this review. On gross examination, carcinosarcomas are usually seen as large, bulky masses that fill the uterine cavity. They may also be seen prolapsing through the cervical os. The cut surface of the mass is fleshy and may show areas of cystic change, necrosis, and hemorrhage. Carcinosarcoma is a biphasic malignant tumor, having distinctive malignant epithelial and mesenchymal components [2], as seen in our cases as well. These components were demonstrated by immunohistochemistry (IHC) in our cases. Pan-cytokeratin was used to demonstrate the epithelial component (Figure 7), while vimentin was used to demonstrate the mesenchymal component in our cases (Figure 8).

Uterine sarcomas usually have a nonspecific clinical presentation. They generally present as a fast-growing pelvic mass and may be associated with abdomen pain or vaginal bleeding [2], as seen in most of the cases in the present study.

Endometrial stromal sarcomas are an uncommon group of malignant tumors that constitute about 10% of all uterine sarcomas but only 0.2% of all cancers arising from the uterus [9]. Taking into consideration the mitotic activity and vascular invasion and prognosis, endometrial stromal tumors may be divided into three categories: endometrial stromal nodule, low-grade endometrial stromal sarcoma, and high-grade endometrial stromal sarcoma. Endometrial stromal sarcomas are common in females in the age range of 45-50 years, as seen in our study as well. However, it can also affect young females. Endometrial stromal sarcomas may be misdiagnosed as a multilocular ovarian cyst or as leiomyoma [9], due to its well-differentiated histomorphology, low mitotic count, mild nuclear atypia and no to minimal necrosis seen in these tumors, as seen in one of our cases, where low-grade endometrial stromal sarcoma was misdiagnosed as leiomyoma during frozen section analysis. However, the subsequent histopathological examination showed a low-grade endometrial stromal sarcoma. The misdiagnosis was due to sampling error during frozen section analysis. The standard treatment for females diagnosed with endometrial stromal sarcoma is total abdominal hysterectomy with bilateral salpingo-oophorectomy. Debulking is generally done in the presence of an extrauterine tumor. The role of chemotherapy in endometrial stromal sarcoma is not very clear. However, low-grade endometrial stromal sarcomas often respond to hormonal therapy, due to the presence of high levels of progesterone receptors [10].

Leiomyosarcomas most commonly arise de novo. However, rarely, they may be seen arising from a sarcomatous transformation in a benign leiomyoma, seen in about 0.2% of the cases. They have an aggressive clinical course, with a five-year survival rate of 18.8%-68%, depending on the stage at the time of diagnosis. Most are seen in older females who are more than 40 years of age, with a median age of around 60 years [2]. Similarly, even in the present study, all females diagnosed with leiomyosarcoma were in their sixth decade. Just like other uterine sarcomas, they also present with abnormal bleeding per vaginum, palpable mass in the pelvis, and abdomen pain [2], which was seen as the presenting symptom in our study as well. Predicting the behavior of the smooth muscle tumors of the uterus using light microscopic features alone is difficult [11]. Bell et al. [12] proposed a classification system based on nuclear atypia, tumor necrosis, and mitotic activity, which was widely accepted, as it was helpful in predicting the clinical outcome in most lesions [11]. However, the mitotic rate thresholds vary in the different subtypes of leiomyosarcoma [13].

On comparison of the present study with various other studies done on uterine sarcomas, the most common age group of females affected with uterine sarcomas in our study was 61-70 years of age. However, in contrast to the present study, the most common age group of females affected with uterine sarcoma in the studies done by Alukal et al. [2] and Cho et al. [14] was 40-49 years and 47.1±10.5 years of age, respectively. Carcinosarcoma was seen in females more than 60 years of age in our study, which was similar to the observations of the studies done by Alukal et al. [2] and Nordal and Thoresen [15]. All females diagnosed with endometrial stromal sarcoma were less than 50 years of age, which was similar to the observations of Alukal et al. [2]. On the contrary, in the study done by Nordal and Thoresen, the most common age group of females diagnosed with endometrial stromal sarcoma was 50-64 years of age [15]. Postmenopausal females were mostly affected with uterine sarcoma in our study and in the study done by Alukal et al. [2]. However, in the study done by Cho et al., premenopausal females were mostly affected [14].

In the present study, the most common histological subtype of sarcomatous transformations in the uterus was carcinosarcoma, which was similar to the observations seen in the studies conducted by Alukal et al. [2] and Wu et al. [8]. But in the studies conducted by Cho et al. [14] and Nordal and Thoresen [15], the most common subtype was endometrial stromal sarcoma and leiomyosarcoma, respectively. The most common presenting feature seen in the females affected with uterine sarcoma in the present study was increased bleeding per vaginum and abdomen pain, which was similar to the observations made in the studies conducted by Alukal et al. [2] and Wu et al. [8]. In the present study, most of the females diagnosed with uterine sarcomas presented at stage I of the disease, except for two carcinosarcoma patients, who presented at stage III of the disease, and one high-grade endometrial stromal sarcoma patient, who presented at stage II of the disease. Similarly, in the studies done by Cho et al. [14] and Nordal and Thoresen [15], most patients presented at stage I of the disease. In the study conducted by Alukal et al. as well, most females with endometrial stromal sarcoma and carcinosarcoma presented in stage Ib of the disease; however, the most common stage at presentation of females with leiomyosarcoma was stage IIa (Table 3) [2].

**Table 3:** Comparison between the various studies ESS, endometrial stromal sarcoma; FIGO, International Federation of Gynecology and Obstetrics

	Present study	Alukal et al. [2]	Cho et al. [14]	Nordal and Thoresen [15]	Wu et al. [8]
Age	61-70 years	40-49 years	47.1±10.5	-	-
Carcinosarcoma	>60 years	≥60 years	-	55-74 years	-
ESS	<50 years	40-49 years	-	50-64 years	-
Most common group involved	Postmenopausal females	Postmenopausal females	Premenopausal females	-	
Most common histological type seen	Carcinosarcoma	Carcinosarcoma	Endometrial stromal sarcoma	Leiomyosarcoma	Carcinosarcoma
Presenting feature	Increased bleeding per vaginum and abdomen pain	Heavy menstrual bleeding and anemia	-	-	Abdomen pain and abnormal uterine bleeding
Stage at presentation	Mostly stage Ⅰ	Carcinosarcoma, stage Ⅰb; leiomyosarcoma, stage Ⅱa; ESS, stage Ⅰb	Mostly FIGO stage Ⅰ sarcoma at diagnosis	Mostly stage Ⅰ	-

The limitation of this present study is the limited number of cases in the study. More number of cases are needed to understand the various presentations and the morphological variations of uterine sarcomas. Molecular studies could not be done for these patients due to the financial constraints faced by the patients.

## Conclusions

Since uterine sarcomas are a rare entity, they are not suitable for screening. To date, there are no effective preoperative diagnostic modalities for uterine sarcomas. Several studies suggested that magnetic resonance imaging (MRI), computed tomography (CT), and serum lactate dehydrogenase (LDH) may be helpful in the diagnosis of uterine sarcomas. However, considering the cost-effectiveness, those diagnostic tools cannot be applied to all patients with uterine masses in a developing country such as India. Thus, surgery, followed by histopathological examination, is the best diagnostic option available. The prognosis for females with uterine sarcoma depends on the extent of the disease at the time of diagnosis and the mitotic index noted. Further studies on uterine sarcomas are needed for a better understanding of the disease.

## References

[1] McMeekin DS (2012). Sarcoma of the uterus. Clinical gynecologic oncology.

[2] Alukal AT, Shankar SB (2019). Clinicopathological spectrum of uterine sarcoma. J Med Sci Clin Res.

[3] Kobayashi H, Uekuri C, Akasaka J, Ito F, Shigemitsu A, Koike N, Shigetomi H (2013). The biology of uterine sarcomas: a review and update. Mol Clin Oncol.

[4] Moskovic E, MacSweeney E, Law M, Price A (1993). Survival, patterns of spread and prognostic factors in uterine sarcoma: a study of 76 patients. Br J Radiol.

[5] Roberts ME, Aynardi JT, Chu CS (2018). Uterine leiomyosarcoma: a review of the literature and update on management options. Gynecol Oncol.

[6] Momeni-Boroujeni A, Chiang S (2020). Uterine mesenchymal tumours: recent advances. Histopathology.

[7] Ferguson SE, Tornos C, Hummer A, Barakat RR, Soslow RA (2007). Prognostic features of surgical stage I uterine carcinosarcoma. Am J Surg Pathol.

[8] Wu TI, Yen TC, Lai CH (2011). Clinical presentation and diagnosis of uterine sarcoma, including imaging. Best Pract Res Clin Obstet Gynaecol.

[9] Berceanu S, Pătraşcu AN, Berceanu C, Tica AA, Bădulescu A (2008). Endometrial stromal sarcoma: clinico-pathological report of four cases and review of the literature. Rom J Morphol Embryol.

[10] Bodner K, Bodner-Adler B, Obermair A (2001). Prognostic parameters in endometrial stromal sarcoma: a clinicopathologic study in 31 patients. Gynecol Oncol.

[11] Calsteren K, Debiec-Rychter M, Moerman P (2005). Clinicopathologic markers of uterine leiomyosarcoma originating from smooth muscle tumors of low malignancy. Eur Clinics Obstet Gynecol.

[12] Bell SW, Kempson RL, Hendrickson MR (1994). Problematic uterine smooth muscle neoplasms: a clinicopathologic study of 213 cases. Am J Surg Pathol.

[13] Oliva E (2016). Practical issues in uterine pathology from banal to bewildering: the remarkable spectrum of smooth muscle neoplasia. Mod Pathol.

[14] Cho HY, Kim K, Kim YB, No JH (2016). Differential diagnosis between uterine sarcoma and leiomyoma using preoperative clinical characteristics. J Obstet Gynaecol Res.

[15] Nordal RR, Thoresen SØ (1997). Uterine sarcomas in Norway 1956-1992: incidence, survival and mortality. Eur J Cancer.

